# Supplementary material for: From sequence to enzyme mechanism using multi-label machine learning
Source: BMC Bioinformatics. 2014 May 19;15:150. doi: 10.1186/1471-2105-15-150 (PMC4229970; doi:10.1186/1471-2105-15-150)
Supplement: Additional file 2 — Java code of ml2db. Additional file ml2db_code.tar.gz contains the Java source code to run the multi-label machine learning experiments and save the results to database. The code’s Javadoc is included. [file 1471-2105-15-150-S2.zip › additional file 2/ml2db/utils/doc/uk/ac/standrews/utils/main/stringutils/package-use.html]

Uses of Package uk.ac.standrews.utils.main.stringutils


JavaScript is disabled on your browser.


- Overview
- Package
- Class
- Use
- Tree
- Deprecated
- Index
- Help

- Prev
- Next

- Frames
- No Frames

- All Classes

# Uses of Package uk.ac.standrews.utils.main.stringutils

- Packages that use uk.ac.standrews.utils.main.stringutils

  | Package | Description |
  | --- | --- |
  |  |  |
  | --- | --- |
  | uk.ac.standrews.utils.main |  |
  | uk.ac.standrews.utils.main.maputils |  |
  | uk.ac.standrews.utils.main.stringutils |  |
- Classes in uk.ac.standrews.utils.main.stringutils used by uk.ac.standrews.utils.main

  Class and Description

  Pool

  Class
- Classes in uk.ac.standrews.utils.main.stringutils used by uk.ac.standrews.utils.main.maputils

  Class and Description

  StringPool

  Class
- Classes in uk.ac.standrews.utils.main.stringutils used by uk.ac.standrews.utils.main.stringutils

  Class and Description

  Pool

  Class

- Overview
- Package
- Class
- Use
- Tree
- Deprecated
- Index
- Help

- Prev
- Next

- Frames
- No Frames

- All Classes
